# Refugees from Ukraine receiving antiretroviral therapy in destination countries and territories of the World Health Organization European Region, including EU/EEA countries, February 2022 to March 2023

**DOI:** 10.2807/1560-7917.ES.2024.29.24.2300567

**Published:** 2024-06-13

**Authors:** Giorgi Kuchukhidze, Machiko Otani, Stela Bivol, Teymur Noori, on behalf of the ECDC/WHO HIV Surveillance network

**Affiliations:** 1 WHO Regional Office for Europe, Copenhagen, Denmark; 2 European Centre for Disease Prevention and Control (ECDC), Stockholm, Sweden; 3 The members of the ECDC/WHO HIV Surveillance network are acknowledged at the end of the article

**Keywords:** ART, HIV, AIDS, Ukraine, Europe

## Abstract

Between the start of the Russian Federation’s invasion of Ukraine on 24 February 2022 and May 2023, more than 8 million individuals have been displaced from Ukraine. Ukraine has the second-largest HIV epidemic in the World Health Organization (WHO) European Region. From a humanitarian and public health perspective it is critical that Ukrainian refugees living with or at risk of HIV have access to testing, treatment and healthcare in their destination country. To gain better insight on the number of refugees from Ukraine receiving antiretroviral therapy (ART) in destination countries, the WHO Regional Office for Europe and the European Centre for Disease Prevention and Control conducted three surveys in July 2022, November 2022 and March 2023. Among 39 countries that responded to at least one survey, 31 had information on the number of refugees from Ukraine receiving ART in their country. A total of 6,519 refugees (1.5 per 1,000 refugees) received ART, lower than previous estimates by WHO, ECDC and partners of between 0.16% and 1.0%. This discrepancy may suggest a substantial number of undiagnosed and/or diagnosed but untreated HIV infections. Improving access to healthcare for people living with HIV among refugees from Ukraine is vital to ensure quality care.

## Background

Since the start of the Russian Federation’s invasion of Ukraine on 24 February 2022, over 8 million people have been displaced from Ukraine. The countries hosting the largest number of refugees from Ukraine are Poland (1,583,563), Germany (922,657), Czechia (504,107), the United Kingdom (201,000), Spain (173,829) and Italy (173,213) [1].

Ukraine has the second-largest HIV epidemic in the World Health Organization (WHO) European Region [2,3]. Thus, from a humanitarian and public health perspective, it is vital that people living with HIV (PLHIV) and/or at risk for HIV who are displaced from Ukraine have access to HIV testing, treatment, and care services in destination countries [4,5].

In 2021, it was estimated that around 240,000 people were living with HIV in Ukraine, equivalent to 0.6% of the population, of which 75% knew their HIV status [2]. Antiretroviral therapy (ART) was provided to ca 130,000 PLHIV (54% of all PLHIV), including 2,700 children [6]. The approach to HIV care in Ukraine follows WHO guidelines. Antiretroviral therapy is provided to all PLHIV, regardless of CD4^+^ cell count, using a standardised regimen of tenofovir, lamivudine (or emtricitabine), and dolutegravir (TLD) as first-line regimen unless there are medical contraindications [7]. Over 80% of adults receiving ART in Ukraine are given this regimen [5].

At the start of the war, there were several attempts to estimate the number of PLHIV among refugees from Ukraine in destination countries to facilitate health services and medication supplies [8,9]. In all cases, it was estimated that refugees would be representative of the Ukrainian population of women, children, and men over 60 years old, as those groups are officially allowed to leave the country. Based on previous estimates, a considerable percentage of refugees would be PLHIV (estimated between 0.16% and 1.0%) [9]. However, the actual number of people living with HIV among refugees from Ukraine remains unknown and countries need to plan their health services with gross estimates for a relatively mobile population with uncertain development.

## WHO/ECDC survey on refugees from Ukraine receiving antiretroviral therapy in European countries

The WHO Regional Office for Europe and the European Centre for Disease Prevention and Control (ECDC) conducted three surveys in July 2022, November 2022 and March 2023 to investigate the number of refugees from Ukraine receiving ART in destination countries. Online surveys using Microsoft forms were sent to the operational contact points (which are designated by national authorities to report HIV/AIDS data to the WHO and ECDC) for HIV/AIDS surveillance in European Union and European Economic Area (EU/EEA) countries and HIV/AIDS surveillance focal points in non-EU/EEA countries of the WHO European Region and Kosovo*. Liechtenstein is an EEA country but not a WHO European Region country. The questions included: (i) name of the respondent; (ii) country; (iii) email address; (iv) availability of information on the number of refugees from Ukraine receiving ART in their country (if available, respondents were asked if they have national data or data from selected locations/parts of the country); (v) number of Ukrainian refugees receiving ART; and (vi) the date this information was available. In the survey sent out in March 2023, respondents were asked to share any challenges they faced in providing access to HIV care and treatment to refugees from Ukraine (the questions from this survey are presented in the Supplementary material). Here we summarise the results of the series of surveys and put the findings into a public health policy-relevant perspective.

## Survey results

Of 55 WHO European Region countries and territories (including EU/EEA countries and Kosovo*), 39 responded to at least one of the three surveys. Twenty-eight countries provided national data and three (Austria, France and Slovakia) provided sub-regional data. As of May 2023, the 28 countries that provided national data hosted 4.2 million of 5.1 million refugees registered for temporary protection or similar national protection schemes in Europe [1]. Eleven and nine countries reported their data in two and three surveys, respectively. Eight countries did not have information on the number of refugees from Ukraine receiving ART. The Table summarises the survey results.

**Table:** Refugees from Ukraine receiving antiretroviral therapy in destination countries of the World Health Organization European Region, including EU/EEA countries (n = 31 countries, latest available data)

Country	PLHIV receiving ART^a^	Number of refugees registered for temporary protection^b^	PLHIV receiving ART per 1,000 refugees	As at	Data availability
Poland	3,150	1,583,563	1.99	March 2023	Countrywide
Germany	896	922,657	0.97	July 2022	Countrywide
Czechia	635	504,107	1.26	March 2023	Countrywide
The Netherlands	245	89,730	2.73	March 2023	Countrywide
Republic of Moldova	185	107,480	1.72	October 2022	Countrywide
Ireland	174	80,085	2.17	December 2022	Countrywide
Romania	125	126,711	0.99	February 2023	Countrywide
Estonia	108	44,739	2.41	October 2022	Countrywide
Lithuania	105	76,540	1.37	December 2022	Countrywide
Norway	102	45,238	2.25	March 2023	Countrywide
Israel	94	NA	NA	November 2022	Countrywide
Sweden	92^c^	53,957	1.71	March 2023	Countrywide
Slovakia	90	114,192	0.79	March 2023	Selected locations^d^
Austria	81	95,993	0.84	February 2023	Selected locations^d^
Bulgaria	74	156,208	0.47	March 2023	Countrywide
Hungary	70	35,030	2.00	November 2022	Countrywide
Belarus	65	22,411	2.90	March 2023	Countrywide
United Kingdom	60	201,000	0.30	March 2023	Countrywide
France	46	118,994	0.39	November 2022	Selected locations^d^
Greece	41	22,704	1.81	February 2023	Countrywide
Croatia	25	21,640	1.16	February 2023	Countrywide
Luxembourg	16	6,756	2.37	February 2023	Countrywide
Iceland	10	2,674	3.74	February 2023	Countrywide
Slovenia	10	8 885^e^	1.13	March 2023	Countrywide
Cyprus	9	21,842	0.41	February 2023	Countrywide
Malta	5	1,744	2.87	March 2023	Countrywide
Azerbaijan	2	5,031	0.40	February 2023	Countrywide
Armenia	1	559	1.79	February 2023	Countrywide
Liechtenstein	1	536	1.87	February 2023	Countrywide
Montenegro	1	8,298	0.12	February 2023	Countrywide
Tajikistan	1	NA	NA	November 2022	Countrywide
Total	6,519	4,150,125^f^	1.57		

ART: antiretroviral therapy; NA: not available; PLHIV: people living with HIV.

^a^ Source: WHO/ECDC survey on refugees from Ukraine receiving ART in European countries, July 2022, November 2022 and March 2023.

^b^ Source: UNHCR (https://data.unhcr.org/en/situations/ukraine) as at May 2023.

^c^ Source: Quality Register InfCareHiv.

^d^ Reported from selected locations/parts of the country.

^e^ As at March 2023.

^f^ Excluding the countries which reported data from selected locations.

In total, 6,519 refugees from Ukraine received ART in the WHO European Region and Lichtenstein since the start of the war. These included those who knew their HIV status and were receiving ART before arriving in their destination country, as well as those who were tested, diagnosed and received ART after arrival in their destination country. The largest absolute numbers of refugees receiving ART were reported from Poland, Germany and Czechia. To estimate the number of PLHIV receiving ART per 1,000 refugees from Ukraine, we used the United Nations Refugee Agency (UNHCR) data on refugees available as at 4 May 2023, which includes the number of refugees officially registered for temporary protection [1]. The overall number of PLHIV receiving ART was 1.6 cases per 1,000 refugees, with nine countries reporting over 2 per 1,000 refugees.

We examined changes over time using data from all countries that reported countrywide data in both surveys conducted in July 2022 and March 2023 (Figure). Among 11 countries, nine reported the number of PLHIV receiving ART in the November 2022 survey. To provide more details on changes, we included data from the November 2022 survey, if available. Ten of the 11 countries experienced an increase in the number of PLHIV receiving ART. In total, there was a 74% increase in the number of PLHIV receiving ART among refugees from Ukraine from July 2022 (2,570) to March 2023 (4,462). While the increase in Poland was the highest in terms of absolute numbers (increased by 1,136 cases), other countries also experienced a substantial change relative to the numbers registered in the first survey (Croatia 47%, Czechia 131%, Greece 95%, Lithuania 300%).

**Figure:**
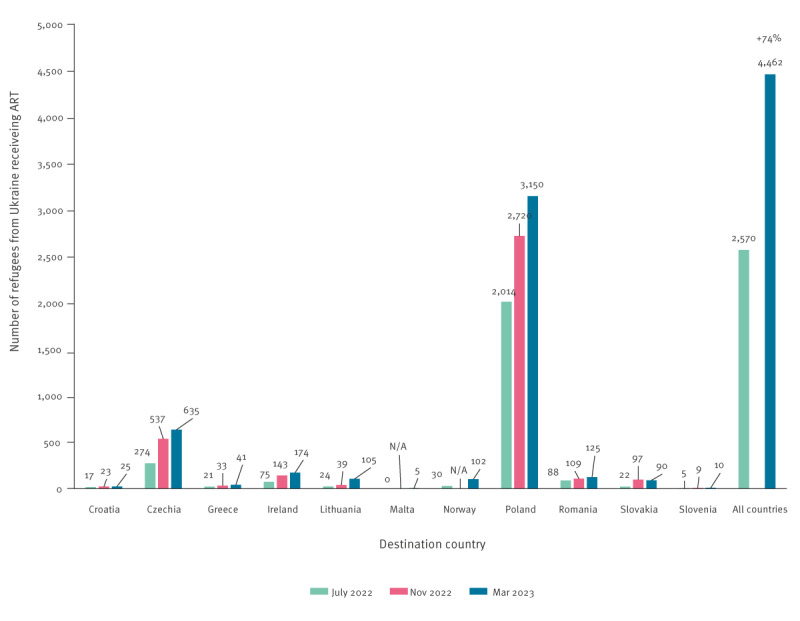
Change over time in the number of refugees from Ukraine receiving antiretroviral therapy in destination countries, July 2022, November 2022 and March 2023 (n = 11 countries)

ART: antiretroviral therapy.

Data are based on the assumption that all refugees from Ukraine receiving antiretroviral therapy in July 2022 remained in the same destination country in March 2023.

While most respondents did not report any challenges in providing access to HIV care and treatment, four countries reported some issues. These included the availability of information on treatment history; language barriers; onward migration and loss-to-follow-up of refugees; and a notable increase in the number of people treated in clinics. For example, Poland, hosting the highest numbers of PLHIV from Ukraine in this study, mentioned the lack of access to TLD and the necessity of a switch to other regimens.

## Public health impact and challenges

The number of refugees from Ukraine receiving ART in destination countries varied considerably across the Region. The average number of PLHIV among refugees from Ukraine was slightly lower in the survey results than the lowest range of estimates produced at the start of the war. While 39 of 55 countries replied to the survey, 28 reported nationally representative data and only 25 countries reported data through the March 2023 survey. These 25 countries accounted for 82% of refugees registered for temporary protection or similar national protection schemes in Europe. However, the absolute number of refugees receiving ART in these countries may be underestimated. The lower-than-expected number of PLHIV receiving ART could be due to undiagnosed HIV infections and/or diagnosed but untreated HIV infections. Another caveat is that countries may host refugees who do not wish to receive temporary protection status and live without registration. However, to receive ART, most countries require refugees to register their presence in the destination country. Thus, this does not affect the rates presented in this paper [10].

A systematic mixed studies review looking at barriers and facilitators affecting the HIV care cascade for migrant PLHIV reported experiences of various barriers impeding linkage to care, ranging from the individual to the policy level [11]. Lack of access to HIV testing and treatment in destination countries could have prevented PLHIV from knowing their HIV status and receiving ART. In addition, stigma and discrimination could have also prevented PLHIV from revealing their HIV status to healthcare providers in destination countries. More comprehensive studies may be needed to understand the details of the current situation of PLHIV among refugees from Ukraine. It is also important for PLHIV to have access to other healthcare services, including hepatitis, tuberculosis, and opioid substitution therapy.

The three surveys were conducted over 9 months, providing a unique opportunity to examine changes over time in the Region. However, we did not collect information on drug resistance, which would be important to examine in future studies. Our results showed a notable increase in the number of PLHIV receiving ART among refugees from Ukraine in the WHO European Region, including EU/EEA countries. This suggests that the demand for ART in destination countries has increased over time. As the number of refugees increases, there may be a further increase in demand. Therefore, destination countries need to prepare for more people requiring ART.

Even though most countries did not report any particular challenges in providing access to HIV care and treatment to refugees from Ukraine as at March 2023, it is concerning that the country hosting the largest number of PLHIV from Ukraine, Poland, reporting 5.4 HIV diagnoses per 100,000 population in 2022, which is close to the EU/EEA average of 5.1 per 100,000 population, reported shortages of WHO-recommended antiretrovirals. Although only Poland reported this issue, it is likely other EU/EEA countries may have experienced the same issue since licensed formulations are available only in low- and middle-income countries through the initiative of the Medicines Patent Pool [12]. A recent study on Ukrainian refugees living with HIV in Czechia reported that 30% of PLHIV switched treatment regimens and that the main reason for switching was drug unavailability [13]. This is concerning, considering the increasing number of PLHIV among refugees from Ukraine reported in countries across Europe [14]. As the situation in Ukraine and neighbouring countries continues to change, it is possible that destination countries will experience changes in migration patterns, including a surge in the number of refugees from Ukraine. Thus, current and possible future destination countries need to effectively collect and share information on PLHIV among refugees from Ukraine to optimise the allocation of resources accordingly.

## Conclusions

The number of PLHIV receiving ART among refugees from Ukraine, obtained from the survey data, was lower than previous estimates. This discrepancy may suggest a substantial number of undiagnosed HIV infections, and/or diagnosed but untreated HIV infections. This underlines the need to improve testing strategies, including indicator condition testing, antenatal screening and community-based testing, as recommended by WHO and ECDC guidelines, to help ensure early diagnosis of HIV and linkage to care. Improving access to healthcare services and considering introducing support systems for refugees to address issues related to loss-to-follow-up, language barriers and increased workload are needed to ensure that all PLHIV among refugees from Ukraine are able to access quality care.

## *Note

This designation is without prejudice to positions on status and is in line with United Nations Security Council Resolution 1244/99 and the International Court of Justice Opinion on the Kosovo Declaration of Independence.

## Supplementary Data

137000 Supplement
