# Supplementary material for: Refugees from Ukraine receiving antiretroviral therapy in destination countries and territories of the World Health Organization European Region, including EU/EEA countries, February 2022 to March 2023
Source: Euro Surveill. 2024 Jun 13;29(24):2300567. doi: 10.2807/1560-7917.ES.2024.29.24.2300567 (PMC11177572; doi:10.2807/1560-7917.ES.2024.29.24.2300567)
Supplement: Supplement [file 23-00567_KUCHUKHIDZE_Supplement.pdf]

"This supplementary material is hosted by *Eurosurveillance* as supporting information alongside the article 'Refugees from Ukraine receiving antiretroviral therapy in destination countries and territories of the World Health Organization European Region, including EU/EEA countries, February 2022 to March 2023', on behalf of the authors, who remain responsible for the accuracy and appropriateness of the content. The same standards for ethics, copyright, attributions and permissions as for the article apply. Supplements are not edited by *Eurosurveillance* and the journal is not responsible for the maintenance of any links or email addresses provided therein."

Annex 1

WHO/ECDC survey on refugees from Ukraine receiving ART in destination countries (March 2023)

1. Name*

2. Country*

3. Email Address*

4. Do you have information on the number of refugees from Ukraine on ART in your country? *

   Yes, countrywide data
   Yes, from selected locations/parts of the country
   No [you can skip Q5]

5. Number of Ukrainian refugees on ART; please also indicate the date (e.g., as of March 1st)

6. Have you encountered any challenges in providing access to HIV care and treatment to refugees from Ukraine that you would like to share with us?

*required fields
